# Exploring the experiences of adolescent *primigravidae* during their first antenatal care visit in Gauteng Province

**DOI:** 10.4102/safp.v68i1.6346

**Published:** 2026-08-20

**Authors:** Mpho Mdakane, Xolani Dlamini, Tshiamo N. Ramalepa

**Affiliations:** 1 Department of Nursing Science, School of Healthcare Sciences, Sefako Makgatho Health Sciences University, Tshwane, South Africa; 2 School of Nursing, College of Health Sciences, University of KwaZulu-Natal, Durban, South Africa

**Keywords:** adolescent *primigravidae*, antenatal care, first experience, maternal health, adolescent pregnancy

## Abstract

**Background:**

Adolescent pregnancy remains a public health concern in South Africa, with adolescent *primigravidae* often facing barriers to antenatal care (ANC) access and continuation. Their first encounter with ANC services is pivotal in shaping future engagement, yet many report uncertainty, stigma and poor communication. This study explored the experiences of adolescent *primigravidae* during their first ANC visit in the Ekurhuleni East sub-district, Gauteng province.

**Methods:**

A qualitative, exploratory, and descriptive design was utilised. Sixteen adolescent *primigravidae* between the ages of 10 and 19 years were purposively chosen from designated ANC clinics and a district hospital in the Ekurhuleni East sub-district of Gauteng Province. Data were gathered through semi-structured interviews, transcribed verbatim and analysed using Creswell’s six-step content analysis framework.

**Results:**

Five themes emerged: (1) Initial ANC clinic visits and uncertainty; (2) negative and positive interactions with the midwives; (3) age-related concerns in the ANC settings; (4) long waiting times; and (5) information and education gaps. While many participants felt hopeful and intended to continue ANC visits, some expressed uncertainty, influenced by judgement and social stigma within the ANC setting.

**Conclusion:**

The first ANC is a critical window for engagement. The experiences shared emphasised the significance of respectful interactions with providers, effective communication, and an environment that is adolescent friendly and supportive in shaping their views on ANC and utilisation.

**Contribution:**

The study offers insight into adolescent-centred ANC improvements and informs recommendations for midwifery practice, health education, and policy development.

## Introduction

Adolescent pregnancy remains a pressing public health concern in South Africa, with wide-ranging social, economic, and health implications for both the adolescent mother and her child. Globally, adolescent girls aged 10–19 years face higher risks of pregnancy-related complications and maternal mortality compared to older women.^1^ In the South African context, adolescent pregnancies are linked to increased school dropout rates, unemployment, dependency on social support systems, and exposure to stigma and discrimination.^2^ As a result, the National Department of Health classifies pregnancies in this age group as high-risk, mandating early booking and consistent antenatal care (ANC) attendance.^3^ However, despite these policy directives, early ANC registration among adolescents remains suboptimal, especially in urban and peri-urban regions such as Ekurhuleni in Gauteng Province.^4^

Adolescents often delay seeking care because of fears of judgement, limited understanding of pregnancy, and negative interactions with healthcare providers.^5^ These barriers can undermine the benefits of ANC, such as timely identification of complications, health education, and interventions to prevent mother-to-child transmission of human immunodeficiency virus (HIV). Understanding the experiences of adolescent *primigravidae* during their first ANC visit is essential for informing service delivery that is responsive to the specific needs of this vulnerable population. Existing literature has predominantly explored ANC attendance from demographic or quantitative perspectives, focusing on statistical correlates such as age, income level or educational attainment.^6,7^ Although these studies are valuable, they often overlook the nuanced, subjective experiences that shape how adolescents perceive and engage with ANC services.

Limited qualitative research exists that centres on the first ANC visit from the perspective of adolescent *primigravidae* in the South African context. Studies conducted in Cape Town indicate that adolescents may encounter dismissive attitudes, breaches of confidentiality, and inadequate communication from healthcare providers during ANC visits, which can discourage follow-up attendance.^8^ However, research focusing specifically on the first ANC visit, arguably the most critical encounter for establishing trust and continuity of care, remains scarce.

This study seeks to address that gap by exploring adolescent *primigravidae’s* experiences during their initial ANC visit in designated ANC clinics and a district hospital in Ekurhuleni East sub-district. By foregrounding adolescents’ *primigravidae* voices, the study contributes original qualitative insights that can inform the design of adolescent-friendly ANC policies and training for healthcare workers.

This study was underpinned by the Health Belief Model (HBM), which is widely used to explain and predict health-related behaviours. The HBM posits that an individual’s decision to engage in health-seeking behaviours is influenced by perceived susceptibility to a health problem, perceived severity of the condition, perceived benefits of taking action, perceived barriers to action, cues to action, and self-efficacy.^9^ This model is particularly relevant in understanding adolescents’ health behaviour, as it provides a lens through which to explore why some pregnant adolescents attend ANC services, while others delay or avoid them. In this study, the HBM was used to guide data collection and analysis by framing participants’ motivations, fears, and perceived outcomes associated with the first ANC visit.

The study aimed to explore the experiences of adolescent *primigravidae* during their first ANC visit at designated ANC clinics and a district hospital in the Ekurhuleni East sub-district of Gauteng Province.

## Research methods and design

This study used an exploratory-descriptive qualitative research design to examine the experiences of adolescent *primigravidae* during their first ANC visit in the Ekurhuleni East sub-district of Gauteng Province. Qualitative research was appropriate for this study because the researchers were interested in the subjective narratives from the participants’ experiences regarding their first antenatal visit. An exploratory and descriptive design was suitable because of the limited existing literature on this subject and allowed for an in-depth understanding of personal experiences from the participants’ perspectives. The study was conducted in the Ekurhuleni East sub-district of the City of Ekurhuleni Metropolitan Municipality, Gauteng Province, South Africa. This sub-district includes a combination of urban and peri-urban communities and is served by a network of public healthcare facilities that provide Basic Antenatal Care (BANC) services. Data were collected from two primary care clinics and one district hospital, all of which were purposively selected because of their high caseloads of adolescent pregnancies.

### Population and sampling

The study population consisted of adolescent girls aged 10–19 years who were pregnant for the first time and attending their first ANC visit at designated ANC clinics and a district hospital. Inclusion criteria were adolescents aged 10–19, pregnant for the first time, had attended only one ANC visit and were able to communicate in English or isiZulu. Exclusion criteria included adolescents with severe physical or mental illness, those receiving care outside the sub-district or undergoing psychological treatment. Non-probability purposive sampling was used to select participants likely to provide rich and relevant data. Adolescent *primigravidae* who were visiting the ANC unit for the first time were interviewed just after their consultation or those who were coming for the second time just before their consultation were selected because they were deemed knowledgeable since they had their first visit experience. The sample size for the study was 16 participants, which was determined by saturation of data. Data saturation occurred during the 13th interview, and then the researchers conducted three additional interviews to confirm data saturation.^10^

### Data collection methods

Data were collected using semi-structured, face-to-face interviews held in private consultation rooms or boardrooms at the designated ANC clinics and a district hospital. Interviews occurred immediately after ANC visits or on their next visit just before consultation with the midwives to allow participants to reflect on their recent experiences. An interview guide was developed based on the study’s theoretical framework, literature review, and research objectives. Section A gathered demographic information; Section B contained open-ended questions such as: ‘Could you please tell me about your experiences regarding your first antenatal care visit?’ and ‘Could you please tell me about your experiences regarding the quality of your first antenatal care visit?’

Recruitment of adolescent *primigravidae* was conducted in collaboration with facility managers, with assistance from their booking records. Posters were placed on clinic notice boards, and the researcher introduced the study during routine health education sessions. Eligible participants were approached after these sessions. Adolescents aged 18 and older provided written informed consent. For those under 18, parental or guardian consent and adolescent assent were obtained. Interviews lasted approximately 20 to 30 min and were conducted in English, isiZulu, or a combination, based on participant preference. All the data collection forms were translated into isiZulu by the researcher. Interviews were audio-recorded with permission and transcribed verbatim. Each participant was assigned a unique code to ensure confidentiality. A pre-test of the interview guide was conducted to ensure that the interview guide collected relevant information and served the right purpose. The first three interviews were included in the pre-test of the data collection tool, and there were no errors or issues during the pre-test. The researchers then included the pre-test interviews in the main data for this study.

### Data analysis

Qualitative content analysis by Creswell was employed to identify patterns and themes in the data.^11,12^ Creswell’s six-step data analysis approach was followed: transcription and familiarisation, initial coding, development of a coding framework, application of codes, identification of themes, and interpretation.^13^ In the initial phase, the researchers systematically prepared and organised the data by transcribing all interviews verbatim to support thorough analysis. These verbatim accounts were compiled into formal transcripts. Once transcription was completed, the data were refined by correcting grammatical and language errors. Segments of the transcripts that were not relevant to the analysis, such as brief, insignificant phrases and the researchers’ own words, were clearly marked. The researchers carefully reviewed and reread each transcript to gain an overall understanding of the tone, central ideas, and general meaning of the data. This review process was repeated to ensure clarity and depth of comprehension. To further enhance understanding and support the coding process, margin notes were recorded to capture emerging insights and interpretations.

During the third phase, coding commenced to generate meaning from the data. The transcripts were organised into related segments, and similar words or phrases were assigned specific letter combinations as codes to enable consistent tracking of recurring information across interviews. As analysis progressed, multiple codes emerged, with some appearing repeatedly across different transcripts. These codes were subsequently grouped and labelled, leading to the development of detailed sub-themes. The sub-themes were then clustered into broader themes. In the final stage, the themes were refined and consolidated to ensure coherence, depth, and meaningful representation of the lived experiences of adolescent *primigravidae* during their first antenatal visit.

All the data transcripts and audio recordings were sent to an independent coder for further analysis and validation.^14^ An independent coder reviewed a subset of transcripts to enhance credibility. A consensus meeting was held to resolve discrepancies and agree on the final codebook. The independent coder and researchers then agreed on the final themes and sub-themes during the consensus meeting.

### Ethical considerations

Ethical clearance to conduct this study was obtained from the Sefako Makgatho University Research Ethics Committee (No. SMUREC/H/24/2024:PG).

## Results

The study recruited adolescent *primigravidae* attending their first ANC visit. Although 15–25 participants were anticipated, data saturation was reached after 13 interviews, with three additional interviews conducted to confirm no new information. The final sample included 16 adolescents. The participants were between the ages of 14 and 18 years, with the youngest being 13 years and the oldest 18 years. Fifteen adolescents were still in school doing Grades 9, 10 and 11, while one 18-year-old was employed as a waitress. Table 1 illustrates their demographic details.

**TABLE 1:** Participants’ demographic information.

Participant	Age (years)	Occupation or Grade	Gestational age (weeks)	Healthcare facility
1	18	Waitress	30	Facility 3
2	16	Grade 10	30	Facility 1
3	14	Grade 9	28	Facility 1
4	16	Grade 10	26	Facility 1
5	17	Grade 11	32	Facility 3
6	18	Grade 11	29	Facility 1
7	14	Grade 9	26	Facility 2
8	13	Grade 8	32	Facility 1
9	18	Grade 11	33	Facility 1
10	16	Grade10	24	Facility 2
11	14	Grade 8	23	Facility 1
12	16	Grade 10	30	Facility 1
13	15	Grade 10	30	Facility 3
14	17	Grade 11	32	Facility 1
15	14	Grade 8	28	Facility 2
16	15	Grade 10	28	Facility 3

Participants demonstrated a clear pattern of late booking for ANC, mainly occurring between 23 and 33 weeks of gestation. This postponement in initiating ANC indicates obstacles to utilising services early, such as fear of stigma, worries about confidentiality, keeping the pregnancy hidden among school-aged adolescents, and a lack of awareness or access to ANC. Overall, these results highlight the necessity for ANC that are adolescent-friendly and focused on interventions to encourage earlier attendance at ANC. To protect the identities of participants and ensure confidentiality, participant number, age, gender and occupation were used to identify the participants.^15^

### Theme 1: Initial antenatal care visits and uncertainty

Adolescent participants described their first ANC visit as marked by uncertainty, anxiety and vulnerability. Many arrived with little knowledge of what to expect, unfamiliar with clinic procedures and the healthcare environment. For most, this was their first experience being treated as autonomous patients rather than dependents, often without prior guidance from family or community resources. Lengthy waiting times, unclear procedures and a lack of orientation heightened their fear and confusion. Several reported midwives failing to introduce themselves or explain processes, which exacerbated feelings of intimidation and reluctance to ask questions. Conversely, those who encountered friendly and supportive midwives experienced increased confidence and reassurance. The following are some of the voices of the participants:

‘I mean, it was my first time coming here, and I did not know where to go and what to do …’ (Participant 4, 16 years old, Female, Scholar)

‘This place [*antenatal care clinic*] was scary when I first came here’. (Participant 2, 16 years old, Female, Scholar)

Most participants expressed that they felt unprepared and unsure about the purpose of ANC visits and what was expected of them during these consultations. They further indicated that they experienced uncertainty during their first visit. Several participants described the ANC environment, characterised by unfamiliar procedures and a serious atmosphere, as intimidating. As a result, many reported feeling confused and fearful during their initial ANC visit, which may have contributed to delays in seeking ANC.

### Theme 2: Positive and negative interactions with the midwives

The nature of interactions between adolescent *primigravidae* and the midwives emerged as a crucial element influencing the overall experience of the first ANC visit. Participants shared a range of experiences, from uplifting and supportive care to disheartening and judgemental encounters. Participants highlighted the critical role of midwife interactions in shaping their ANC experience. While many recounted supportive, respectful, and patient-centred care that fostered confidence and trust, others described negative encounters involving rudeness, shouting, or patronising attitudes. Negative interactions reinforced feelings of shame and stigma, deterring some from future ANC visits. Overall, positive experiences predominated and were described as empowering and encouraging adherence to care:

‘They [*midwives*] were very kind. Very, very kind. Very helpful’. (Participant 1, 18 years old, Female, Waitress)

‘The overall treatment [*by the midwives*] was alright’. (Participant 7, 14 years old, Female, Scholar)

‘It is alright … They [*midwives*] treat people well’. (Participant 8, 13 years old, Female, Scholar)

‘They [*midwives*] were very kind. Okay. Very, very kind. Very helpful. Like the nurses, the sisters. Even like the patients. They’re also very kind’. (Participant 1, 18 years old, Female, Waitress)

Negative encounters had been minimal, with only a few participants reporting feelings of distress, frustration, or isolation. Nonetheless, one participant’s experience is contradictory to most of the participants as she assimilates her ANC with feelings of dissatisfaction and discomfort, which differed from the generally positive reflections shared by most participants. The quotes were as follows:

‘The sister [*midwife*] I met outside was just rude; I had a mask on because I had the flu. And she said when I talk to boys, I don’t talk that softly’. (Participant 6, 18 years old, Female, Scholar)

‘The environment was okay. You know, it was okay. It’s just that the nurses [*midwives*] do shout, some of them’. (Participant 5, 17 years old, Female, Scholar)

‘It was alright, but they [*midwives*] kept shouting at me. Like, I mean, it was my first time coming here and I did not know where to go and what to do …’ (Participant 4, 16 years old, Female, Scholar)

Overall, the supportive conduct of midwives contributed to a more reassuring first experience of ANC for adolescent *primigravidae*. Participants indicated that their interactions with midwives during ANC consultations included both positive and negative experiences, which influenced their perceptions of ANC and their willingness to utilise these services. Positive interactions fostered trust and confidence, thereby encouraging continued attendance and engagement with ANC services. In contrast, negative encounters caused some participants to feel reluctant or disconnected from the healthcare system, which could potentially discourage them from seeking ANC in the future.

### Theme 3: Age-related concerns

Age-related concerns emerged as a sub-theme that strongly shaped how adolescent *primigravidae* experienced ANC. Participants reported feeling uncomfortable and intimidated in the ANC clinic environment, where most patients were considerably older than they were. They explained that this age difference created both a visible and emotional separation, often leaving them feeling judged, inferior, or reluctant to speak. Many participants indicated that they found it difficult to interact confidently with midwives or other pregnant women, as they feared that their young age might lead to criticism or dismissive attitudes. Adolescents felt out of place and judged in ANC settings dominated by older women. The age gap fostered feelings of alienation and diminished confidence in communicating with healthcare providers. Many perceived ANC clinics as adult-centric environments that did not address their specific needs. Being assigned to high-risk adolescent clinics was unexpected and sometimes unwelcome. Participants also reported stigma and stereotyping because of their young age, which negatively influenced their ANC experience:

‘They told me that it’s because I am too young and I had to see a social worker’. (Participant 7, 14 years old, Female, Scholar)

‘You are still a child, what are you doing with a child? But nurses were like ‘you are so pretty’. (Participant 6, 18 years old, Female, Scholar)

‘They [*the midwives*] told me to start attending here because I am young’. (Participant 8, 13 years old, Female, Scholar)

The participants further reflected that they had often felt labelled or stereotyped because of their age, which had led to experiences of stigma in the ANC settings. They had expressed that ANC services had not always been designed to meet their unique needs, leaving them feeling unsupported and, at times, unwelcome. They added that the first ANC experience had influenced their perceptions of ANC and their overall comfort in seeking ANC.

### Theme 4: Long waiting times

Prolonged waiting times emerged as a barrier to effective ANC for adolescent *primigravidae*, substantially shaping their initial perceptions and overall experiences of ANC services. Prolonged waiting periods were a significant barrier, leading to frustration, fatigue, and increased anxiety. Many adolescents found waiting physically uncomfortable and disruptive to school and household responsibilities. The slow pace often reduced the perceived value of the visit, especially when consultation times were brief. Some participants reported missing classes or exams due to lengthy clinic stays, which discouraged return visits:

‘I felt like it was time-consuming because I ended up spending the whole day here’. (Participant 8, 13 years old, Female, Scholar)

‘We do wait for a long time’. (Participant 10, 16 years old, Female, Scholar)

‘It’s not that bad, but the people that I was following said today it was apparently slow because the doctors came late’. (Participant 4, 16 years old, Female, Scholar)

Participants indicated that they often felt overlooked or undervalued because of the long waiting periods at ANC clinics. This situation increased their anxiety about how they would be treated once they were eventually attended to by the midwives and the doctor. In addition, extended waiting times discouraged some adolescent *primigravidae* from returning for follow-up ANC visits. From a physical perspective, participants reported discomfort such as swelling and back pain caused by sitting for long periods. Long waiting times also disrupted their daily responsibilities, including school attendance and household tasks. Some participants further observed that they had missed classes or examinations because they had spent most of the day at the clinic.

### Theme 5: Information and education gaps

The findings of the study also identified the existence of information and education gaps during the first ANC visits, which somewhat influenced the experiences of the adolescent *primigravidae*. Participants conveyed feelings of being overwhelmed or confused by the medical details shared with them, highlighting that explanations were typically hurried, overly technical, or not suited to their comprehension level. Information provided during the first ANC visit was often overwhelming, technical, and insufficiently tailored to adolescents’ comprehension levels. Many participants felt rushed through explanations and hesitant to ask questions, and sometimes met with reprimands when seeking clarity. Group education sessions were perceived as generic and did not address adolescents’ unique needs. This gap contributed to confusion, anxiety, and misinformation, undermining empowerment and follow-up engagement:

‘I was scared because I did not know that a teenage pregnancy is a risk’. (Participant 7, 14 years old, Female, Scholar)

‘They give us instructions once and leave us to continue, but if you ask questions, they start shouting’. (Participant 4, 16 years old, Female, Scholar)

‘I was told that PrEP will prevent me from contracting HIV, and the implant is good because I am young’. (Participant 3, 14 years old, Female, Scholar)

Participants indicated that limited knowledge had often contributed to hesitation in seeking ANC, which in some cases resulted in unfavourable pregnancy outcomes. Many adolescent *primigravidae* reported that midwives had not always communicated information in a clear or relevant manner, leading to confusion and leaving them inadequately prepared for pregnancy and childbirth. Ineffective communication was identified as a major challenge, making it difficult for participants to understand essential information. In addition, they expressed that limited access to reliable information heightened their anxiety and facilitated the spread of misinformation among them.

## Discussion

This study explored the first ANC experiences of adolescent *primigravidae*, revealing a combination of structural and interpersonal challenges that shaped their perceptions and engagement with ANC. The findings of this study are from the narratives of adolescent *primigravidae*, drawing attention to both structural and interpersonal factors that influenced their perceptions and interactions with ANC services. These results highlight that the initial ANC visit serves as an essential gateway into the maternal healthcare system for the adolescent *primigravidae* and can greatly impact their future involvement with pregnancy care services.

Participants described a range of experiences during their ANC visits that reflected both systemic shortcomings and interpersonal challenges. Notably, a few adolescent *primigravidae* reported positive and supportive encounters with the midwives who were patient, reassuring, and eager to explain pregnancy-related procedures. Respectful and adolescent-sensitive communication may help to reduce some of the worries related to early pregnancy and healthcare participation because these interactions seemed to increase participants’ sense of psychological safety and trust. For adolescents who are often navigating pregnancy for the first time, these supportive interactions can contribute to improved confidence in the midwives and greater willingness to seek continued care.

Commonly reported difficulties included long waiting times, communication that was not adapted to adolescents’ needs, and limited privacy during consultations. These barriers often left adolescent *primigravidae* feeling overlooked, invisible, or even judged for their pregnancies. The lack of privacy and adolescent-sensitive communication is particularly significant, as the adolescent *primigravidae* may have already experienced heightened vulnerability, stigma, or fear when accessing ANC. In such contexts, the absence of supportive communication may reinforce feelings of marginalisation and discourage open dialogue between adolescents and the midwives. Some participants reported confusion about clinical procedures because of inadequate explanations, while others felt intimidated by an ANC environment they perceived as strict and unsupportive. Common issues included long waiting times, a lack of adolescent-specific communication, limited privacy, and feelings of being marginalised within ANC clinics. These factors often led to emotional distress, with participants feeling overlooked or judged for their pregnancy.

A central finding was the influence of midwives’ attitudes on adolescents’ ANC experiences. This emerged as a critical determinant of how adolescents interpreted and navigated their care experiences. While some adolescents described positive interactions that fostered comfort and trust, many reported feeling criticised or dismissed. These negative encounters discouraged further ANC attendance and reinforced stigma. Supportive and empathetic midwives, by contrast, improved adolescents’ willingness to return and engage in care. This finding echoes the study by Erasmus et al.^6^ who found that stigma from healthcare providers undermines adolescents’ maternal health-seeking behaviours. Structural barriers further impacted ANC access. The coexistence of both positive and negative midwives’ behaviours suggests variability in the implementation of adolescent-friendly care principles within ANC services. Addressing this inconsistency may require targeted training and sensitisation of healthcare providers to improve respectful maternity care for the adolescent *primigravidae*.

Financial hardship, long travel distances, and reliance on family support were significant obstacles, especially for adolescents from low-income households. Similarly, it was reported that economic challenges hinder ANC utilisation among adolescents and contribute to poor maternal outcomes.^6^ Structural and logistical challenges further intensified the barriers adolescents encountered when trying to access ANC services. Some participants indicated that financial constraints, transportation problems, and long distances to the ANC clinic hindered their ability to attend scheduled visits. These difficulties were particularly evident among adolescents from low-income households, who often depended on family members for financial support to reach the clinic. Similar observations were reported by Tolossa et al.,^7^ who found that adolescents from economically disadvantaged backgrounds face significant obstacles in accessing ANC and other maternal health services, which can negatively influence both maternal and neonatal health outcomes.

Long waiting times and overcrowding were additional deterrents. Participants felt sidelined in favour of older women, contributing to a belief that ANC services were not designed for adolescents. This perception may contribute to adolescents feeling invisible within maternal health systems that are traditionally structured around adult women. Such experiences can weaken the adolescent *primigravidae’s* sense of belonging within healthcare spaces and may reduce their motivation to engage consistently with ANC services. These findings align with Sewpaul et al.^8^ who highlighted adolescent isolation in ANC settings because of service inefficiencies. However, a few participants indicated that despite these barriers, they still valued the opportunity to receive pregnancy-related information and ANC services. This suggests that the adolescent *primigravidae* recognises the potential benefits of ANC services even when service delivery challenges exist, reinforcing the importance of improving service environments to maximise engagement.

Knowledge gaps also emerged as a key concern. Many adolescents delayed their first ANC visit because of a lack of understanding of its importance or reliance on misinformation. This highlights a need for improved reproductive health education, particularly in early adolescence, as observed by Erasmus et al.^6^ This finding reflects persistent gaps in adolescent reproductive health education and suggests that early pregnancy recognition and timely healthcare seeking may be hindered by inadequate access to reliable health information. Conversely, some participants demonstrated awareness of the benefits of ANC, particularly when they had received guidance from the midwives, family members, or educational sources. These contrasting experiences highlight the potential impact of comprehensive reproductive health education in empowering adolescent *primigravidae* to make informed healthcare decisions. Overall, the study underscores the importance of adolescent-friendly ANC services that prioritise respectful communication, reduce stigma, and address both emotional and structural barriers. Ensuring a positive initial ANC experience may increase ongoing engagement and improve maternal and neonatal outcomes.

### Strengths and limitations

This study was limited by its small, purposively selected sample of 16 adolescent *primigravidae*, which may restrict generalisability. All participants were drawn from urban and peri-urban community facilities in Gauteng Province, and their experiences may differ from those of adolescents in rural or other provincial contexts. The qualitative research approach provided an in-depth understanding of the experiences of adolescent *primigravidae*. However, the findings cannot be generalised to all adolescents because of the context-specific nature of qualitative research and the relatively small sample size. Despite these limitations, the study provides valuable insights into the experiences of adolescent *primigravidae* and highlights important considerations for improving adolescent-friendly ANC services.

### Recommendations

The following are some of the key recommendations for improving adolescent ANC engagement:

- Provision of adolescent-responsive services that respect privacy and support autonomy.
- Training for midwives on empathetic, non-judgemental, age-appropriate communication.
- Structural improvements to reduce waiting times and improve the service environment.
- Community-level interventions to reduce stigma and enhance awareness.
- Inclusion of family and community members in adolescent support systems.
- These findings underscore the need for targeted, multilevel strategies to improve ANC uptake and experience among adolescent *primigravidae*.
- Establish adolescent-friendly ANC spaces that promote confidentiality and dignity.

## Conclusion

This study achieved its aim of exploring the experiences of adolescent *primigravidae* during their first ANC visit at selected facilities in the Ekurhuleni East sub-district, Gauteng Province. The initial ANC encounter plays a pivotal role in shaping adolescents’ future engagement with maternal health services. Adolescent *primigravidae* reported heightened anxiety, fear of judgement, and feelings of stigma during their initial ANC visit, which often delayed their healthcare engagement. Contributing factors included poor communication by midwives, long waiting periods, lack of privacy, and inadequate adolescent-specific services. External influences such as family conflict and societal disapproval added emotional strain and social pressure. Findings revealed that negative experiences are characterised by stigma, poor communication, and systemic inefficiencies; moreover, they can undermine trust and continuity of care. Conversely, respectful, adolescent-friendly interactions foster a sense of safety and motivation for ongoing participation. Improving the quality of first-contact ANC services is therefore essential for enhancing maternal and neonatal outcomes among this vulnerable population.
